# Management of pterygium: our experience and a simplified treatment algorithm

**DOI:** 10.22336/rjo.2024.21

**Published:** 2024

**Authors:** Shreesha Kumar Kodavoor, Ashalyne James Joseph, Shreyas Ramamurthy, Ramamurthy Dandapani

**Affiliations:** * The Eye Foundation, Coimbatore, India

**Keywords:** primary double-head pterygium, recurrence rate, recurrent pterygium, treatment algorithm

## Abstract

**Aim:** To explore various approaches in the management of pterygium and to propose a simplified treatment algorithm for its surgical management.

**Methods:** A retrospective analysis of 9219 eyes was done. Group I included patients with primary single-head pterygium, most undergoing pterygium excision with conjunctival autograft (CAG). CTG-P (Conjunctival tissue graft from pterygium), AMG (Amniotic membrane graft), and inferior CAG were done in the remaining patients in this group in which conventional conjunctival autograft was a relative contraindication. Group II included patients with primary double-head pterygium who underwent vertical/horizontal split CAG, with/without limbal orientation, Inferior + Superior CAG, CTG-P, and CAG + CTG-P. Group III included patients with recurrent single-head pterygium who underwent ER (Extended resection) + LCAG (Limbal conjunctival autograft), LCAG + MMC (Mitomycin-C), CAG + MMC (Mitomycin-C) and CAG. Group IV included patients with recurrent double-head pterygium who underwent split LCAG and CAG + SLET.

**Results:** All the four groups reported a low incidence of pterygium recurrence. Recurrence was observed at a rate of 0.47%, 3.63%, 2.86%, and 7.69% in Group I, Group II, Group III and Group IV respectively.

**Discussion:** We mainly aimed to get minimal recurrence and good cosmetic outcomes. In double-head pterygium, we could achieve good and comparable outcomes with horizontal or vertical split CAG, with or without maintaining limbal orientation. Similarly, Inferior + Superior CAG, CTG-P, CAG+CTG-P, and AMG also showed low recurrence rates. In recurrent pterygium, ER + LCAG/CAG, with/without adjuncts like MMC showed low recurrence rates. Thus, all of these methods were found to be viable options. The main strength of our study, compared to previous studies on pterygium was its large sample size and long duration of follow-up.

**Conclusion:** All the methods we studied had a low recurrence rate. We have formulated a treatment algorithm for pterygium management based on our outcomes.

**Abbreviations:** CAG = Conjunctival autograft, CTG-P = Conjunctival tissue graft from pterygium, ER = Extended resection, MMC = Mitomycin-C

## Introduction

Pterygium is the development of a wing-shaped tissue encroachment over the cornea. Exposure to ultraviolet radiation is one of the major contributing factors. Despite its common nasal occurrence, double-head pterygium is frequently seen in tropical countries [**1**]. Elastotic degeneration of conjunctival collagen is the main histopathological feature [**2**]. 

A crucial aspect of pterygium treatment is to decrease the likelihood of recurrence. Factors like incomplete resection, bare sclera technique, and young age can contribute to pterygium recurrence.

Pterygium surgery has been performed for more than 3000 years since the era of Sushruta [**3**]. Different surgical techniques have been explored, each presenting unique advantages and disadvantages. Conventional CAG (Conjunctival autograft) is widely regarded as the standard of care. The superior bulbar conjunctiva may not be accessible at times or provide adequate coverage for bare scleral defects. Hence, a range of techniques have been explored.

Our study details our approach to managing various forms of pterygium through some procedures. We have developed a treatment algorithm based on our findings to guide the management of different pterygium types.

## Materials and methods

The study encompassed pterygium excision procedures conducted on 9861 eyes between January 2005 and December 2020. Of these, 9219 eyes met the 12-month follow-up requirement and were included in the retrospective analysis. The duration of follow-up varied between 12 months to 15 years. In this study, the data was collected from the hospital’s EMR (Electronic Medical Records). In total, 7663 patients underwent pterygium excision, among which 6107 underwent unilateral and 1556 underwent bilateral pterygium excision. 7602 eyes underwent nasal pterygium excision, whereas 904 eyes underwent temporal pterygium excision. Regarding gender distribution, the study comprised 4201 males and 3462 females. The patients were classified into four groups based on the characteristics of their pterygium.

Group I included 8506 eyes with primary single-head pterygium, Group II included 385 eyes with primary double-head pterygium, Group III included 315 eyes with recurrent single-head pterygium and Group IV included 13 eyes with recurrent double-head pterygium. 328 eyes had recurrent pterygium and 398 eyes had double head pterygium. **Table 1** outlines the various surgical methods employed across the four groups. All surgeries were performed at a tertiary eye hospital in India’s Southern part by a single experienced surgeon.

**Table 1:** Comparison of different methods of pterygium excision in our study

PTERYGIUM	TYPE OF SURGERY	NO. OF EYES	RECURRENCE
Group I - Primary single-head pterygium	CAG (Conjunctival autograft)	8316	0.38% (32/8316)
	CTG-P (Conjunctival tissue graft from pterygium)	90	3.33% (3/90)
	AMG (Amniotic membrane graft)	48	6.25% (3/48)
	Inferior CAG	52	3.84% (2/52)
Group II - Primary double-head pterygium	Vertically split CAG with limbal orientation	180	2.22% (4/180)
	Vertically split CAG without limbal orientation	75	2.67% (2/75)
	Horizontally split CAG	70	4.29% (3/70)
	Inferior + Superior CAG	30	3.33% (1/30)
	CTG-P	18	11.11% (2/18)
	CAG + CTG-P	12	16.67% (2/12)
Group III - Recurrent single-head pterygium	ER (Extended resection) + LCAG (Limbal conjunctival autograft)	280	2.14% (6/280)
	LCAG + MMC (Mitomycin-C)	12	0.89% (1/12)
	CAG	8	12.5% (1/8)
	CAG + MMC	15	6.67% (1/15)
Group IV - Recurrent double-headed pterygium	LCAG	10	10% (1/10)
	CAG + SLET	3	0% (0/3)

A detailed clinical examination and history were taken before surgery. Primary pterygium was classified by the degree of its involvement [**4**]. Patients who were known cases of glaucoma with filtering blebs or glaucoma suspects underwent procedures like CTG-P (Conjunctival tissue graft from pterygium), AMG (Amniotic membrane graft), or Inferior CAG (Conjunctival autograft) [**5**].

Recurrent pterygium was classified by the degree of its involvement [**6**]. Individuals with actual corneal recurrence were operated on.

The protocol for the study was consistent with the ethical standards of the Declaration of Helsinki. The study protocol adhered to the tenets of the Declaration of Helsinki and obtained clearance from the Institutional Ethics Committee. 

*Surgical procedure*

In all the methods, the graft was fixed using fibrin glue, Tisseel (Baxter, Austria).

CAG - A superior or superior-temporal conjunctival graft was dissected. It was then used to cover the defect over the sclera. 

CTG-P - The conjunctival layer from the pterygium was separated. It was then used to cover the defect created by removing the tissue beneath. 

Inferior CAG - Graft tissue was harvested from the inferior conjunctiva. 

ER (Extended resection) + LCAG (limbal conjunctival autograft) - The pterygium tissue was dissected extensively. 1 mm margin of conjunctival tissue and 0.5 mm margin of tenon was also removed. When harvesting the graft, a portion of the cornea was also included. Dissection of 100 microns thickness and 0.5 mm into the cornea was done. This graft, containing stem cells of the limbus, was placed on the defect over the sclera. 

**Fig. 1:**
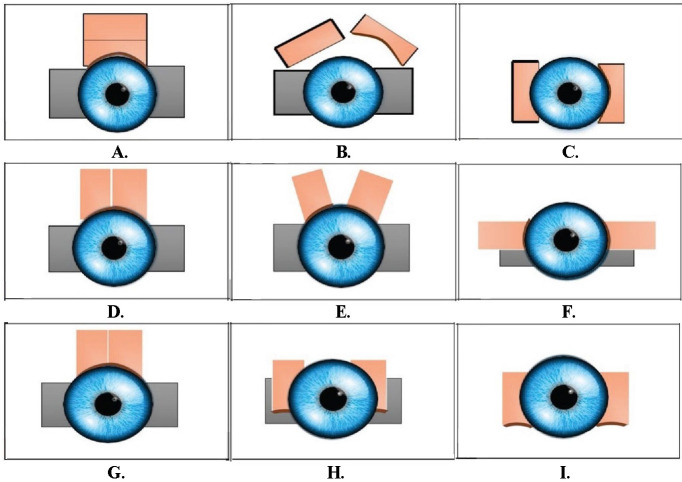
Split CAG. **A.** Adequately sized graft taken from superior conjunctiva; **B.** The graft was cut into two halves horizontally; **C.** Split grafts placed over the two defects; **D.** The superior conjunctival graft is vertically split into two halves; **E.** Limbal orientation is maintained; **F.** Graft may not completely cover bare area; **G.** Superior conjunctival graft is vertically divided into two halves; **H.** The limbal alignment is not upheld; **I.** Graft completely covers the bare area

Horizontally split CAG - Graft tissue was harvested from the superior portion of the conjunctiva. This graft was then divided horizontally to obtain two parts. The split conjunctival autografts were placed over the defects over the sclera on the two sides (**Fig. 1 A-C**).

Vertically split CAG - Graft tissue was harvested from the superior portion of the conjunctiva. This graft was then divided vertically to obtain two parts. The split conjunctival autografts were placed over the defects, the sclera, on the two sides, either with or without limbal orientation (**Fig. 1 D-I**).

Inferior + Superior CAG - One graft was harvested from the superior conjunctiva and one from the inferior. These two separate grafts were placed over the two defects overlying the sclera.

CAG + CTG-P - A thin conjunctival layer was separated from one head of the pterygium. This layer was used to cover the defect overlying the sclera. The second defect was covered by a graft obtained from the superior conjunctiva.

During the postoperative period, the patients were put on steroids in tapering doses, antibiotics, and lubricants. During the postoperative visits, recurrence of pterygium was specifically looked for. 

## Results

A comprehensive analysis was conducted on 9219 eyes with pterygium. The eyes were categorized as follows: 8506 eyes in Group I, 385 eyes in Group II, 315 eyes in Group III, and 13 eyes in Group IV. The minimum period of follow-up was 12 months. The longest period of follow-up was 15 years.

In Group I, 0.47% (40/8506) of eyes showed recurrence. The recurrence rate was low in all the techniques, varying from 0.38% to 6.25%. The recurrence was lowest with CAG (0.38%). Inferior CAG (3.84%) and CTG-P (3.33%) showed comparable recurrence rates. The recurrence rate was highest with AMG (6.25%). 

In Group II, 3.63% (14/385) of eyes showed recurrence. The recurrence rates varied between 2.22% and 16.67%. The recurrence was low and comparable in eyes undergoing vertically split CAG with limbal orientation (2.22%) and lacking limbal orientation (2.67%), horizontally split CAG (4.29%), and inferior + superior CAG (3.33%). Higher recurrence rates were noted with CTG-P (11.11%) and CAG + CTG-P (16.67%). The lower number of cases studied in this sub-group could be due to these higher recurrence rates.

In Group III, 2.86% (9/315) of eyes showed recurrence. The recurrence rates varied between 0.89% and 12.5%. The recurrence was lowest with LCAG + Mitomycin-C (MMC) (0.89%). ER + LCAG (2.14%) and CAG + MMC (6.67%) also showed low recurrence rates. CAG alone showed the highest recurrence rate (12.5%). 

In Group IV, 7.69% (1/13) of eyes showed recurrence. 10% of eyes showed recurrence following split LCAG. Eyes that underwent CAG combined with SLET showed no signs of recurrence.

## Discussion

The key objective of pterygium excision is to ensure minimal recurrence and good cosmetic outcomes. The most widely used method is conjunctival autografting. In our study, we aimed to formulate a treatment algorithm for different types of pterygium. 

In double-headed pterygium, the conjunctival autograft can be split horizontally or vertically; with or without maintaining limbal orientation. All these methods were found to have good outcomes with low and comparable recurrence rates in our study. They also had cosmetically acceptable outcomes. 

In some instances, especially with significant defects over the sclera defects, keeping the graft’s orientation intact on the limbal side may not be feasible [**7**]. We observed no notable variation in the recurrence rates of the different surgical techniques.

An earlier investigation at our institute on a different cohort followed the vertical split CAG method without preserving orientation on the limbal side [**4**]. Only two eyes out of 95 developed recurrence (2.10%). Results from this study also demonstrate that this method is successful. 

Other methods that can be used for double-head pterygium include Superior + inferior CAG, CTG-P, CAG + CTG-P, and AMG. We found that each method exhibited low recurrence rates, making them viable options. 

Few authors studied the management of double-headed pterygium. Maheswari et al. reported no recurrence with the horizontal split CAG method [**1**]. Elhamaky et al. reported no recurrence with the vertical split CAG method [**8**]. The studies mentioned above were limited by their small study population.

Approaches for addressing recurrent pterygium encompass ER with LCAG/CAG, with/without MMC. Each method exhibited low recurrence rates, thus being a viable option.

Mashroof et al. compared the recurrence rates of LCAG and CAG. LCAG was considered the better method (0.95% vs. 10%) [**9**]. Jun et al. compared the recurrence rates of AMG + LCAG and CAG. LCAG was considered the better method (0.95% vs. 10%). AMG + LCAG was considered the better method (8.3% vs. 20%) [**10**].

The utilization of various adjuncts is beneficial in reducing recurrence [**11**]. Complications associated with mitomycin-C include scleral melt, corneal melt, and punctate keratopathy [**12**]. Difficulties related to amniotic membranes are their cost, storage, and availability. The literature also indicates a higher recurrence rate with this method [**13**]. Grafts can be secured using either fibrin glue or sutures. Advantages of fibrin glue include ease of fixation, lesser operation time, and less postoperative discomfort. 

A total of 9219 eyes with pterygium were included in our study. The recurrence rate was 0.47% in Group I, 3.63% in Group II, 2.86% in Group III and 7.69% in Group IV. **Tables 2** and **3** show our study’s outcomes compared to previous studies on double head and recurrent pterygium.

**Table 2:** Analyzing our results alongside existing studies on surgical management of double headed pterygium

AUTHOR	PTERYGIUM	TYPE OF SURGERY	NO. OF EYES	RECURRENCE
Our study	Primary	Vertically split CAG with limbal orientation	180	2.22% (4/180)
		Vertically split CAG without limbal orientation	75	2.67% (2/75)
		Horizontally split CAG	70	4.29% (3/70)
		Inferior + Superior CAG	30	3.33% (1/30)
		CTG-P	18	11.11% (2/18)
		CAG + CTG-P	12	16.67% (2/12)
	Recurrent	LCAG	10	10% (1/10)
		CAG + SLET	3	0% (0/3)
Kodavoor et al. [**4**]	Primary	Vertically split CAG without limbal orientation	95	2.10% (2/95)
Elhamaky et al. [**8**]	Primary	Vertically split CAG; limbal-to-limbal, fibrin glue	15	0% (0/15)
Dwivedi et al. [**14**]	Primary	Horizontally split CAG; alignment not specified, electro-coaptation, sutures	8	12.5% (1/8)
Hirst et al. [**15**]	Primary Recurrent	Sequential PERFECT; 6-month interval	20	0% (0/20)
Maheshwari et al. [**1**]	Primary	Split CAG with limbus limbus orientation on the nasal side	7	0 % (0/7)
Chan et al. [**16**]	Primary Recurrent	CAG; oversized 1 mm, 8-0 polyglactin	32	6.25% (2/32)
	Primary Recurrent	BST (Bare sclera technique) + MMC (0.02%, 5 mins)	32	28.1% (9/32)

**Table 3:** Our study versus other research on recurrent pterygium surgery

AUTHOR	PTERYGIUM	TYPE OF SURGERY	NO. OF EYES	RECURRENCE
Our study	Recurrent single head	ER (Extended resection) + LCAG	280	2.14% (6/280)
		LCAG + MMC (Mitomycin-C)	12	0.89% (1/112)
		CAG	8	12.5% (1/8)
		CAG + MMC	15	6.67% (1/15)
	Recurrent double head	Split LCAG	8	12.5% (1/8)
		CAG + SLET	5	0% (0/5)
Mashfoor et al. [**15**]	Recurrent single head	LCAG	105	1 (0.95%)
		CAG	100	10 (10%)
Lawrence Hirst W et al. [**17**]	Recurrent single head	P.E.R.F.E.C.T.	111	6 (2.7%)
Shimazaki J et al. [**10**]	Recurrent single head	AMG with Limbal CAG	15	3 (20%)
		AMG with CAG	12	1 (8.3%)

**Fig. 2:**
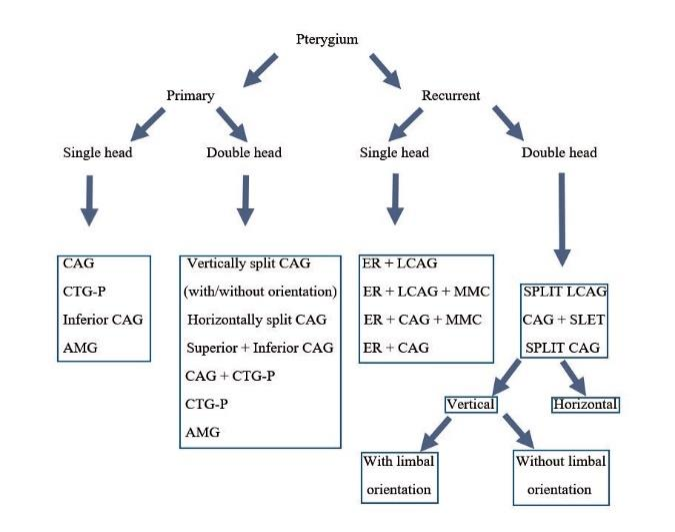
Treatment algorithm for pterygium

The study’s limitation lies in its retrospective nature, while its major strength is the substantial sample population and prolonged follow-up duration. As far as we know, this study is one of the largest-scale assessments comparing the efficacy of various methods in addressing different types of pterygium. Based on our experience, the present study included, we have formulated a treatment algorithm for pterygium management (**Fig. 2**). 

## Conclusion

We mainly focused on comparing the recurrence rates in various surgical methods used in different types of pterygium in our study. All the methods we studied had a low recurrence rate. Based on our outcomes, a treatment algorithm was formulated for pterygium management. An extended follow-up period and a prospective study would give us more robust data on the effectiveness of the various methods.

**Conflict of Interest Statement**

The authors state no conflict of interest.

**Informed Consent and Human and Animal Rights Statement**

Informed consent has been obtained from all individuals included in this study.

**Authorization for the use of human subjects**

Ethical approval: The research related to human use complies with all the relevant national regulations, and institutional policies, as per the tenets of the Helsinki Declaration, and has been approved by the review board of The Eye Foundation, Coimbatore, India (TEF/IEC/2023/032, 09/11/2023).

**Acknowledgments**

None.

**Sources of Funding**

None.

**Disclosures**

None. 
